# Ash clouds temperature estimation. Implication on dilute and concentrated PDCs coupling and topography confinement

**DOI:** 10.1038/s41598-019-42035-x

**Published:** 2019-04-04

**Authors:** A. Pensa, L. Capra, G. Giordano

**Affiliations:** 1 Centro de Geociencias, Universidad Nacional Autónoma de México (UNAM), Querétaro, Mexico; 2 Science Department, Roma Tre University, Roma, Italy

## Abstract

Pyroclastic density currents (PDCs) are among the most hazardous of all volcanic processes in terms of high speeds and unpredictable extent. While concentrated PDCs are usually topographically confined, the dilute counterpart (ash cloud) is able to overrun topographic barriers, with unexpected trajectories posing a high risk for human settlements around the volcano. Here, for the first time, the temperature of an ash could, for a PDC originated during the 11 July, 2015 Volcán de Colima eruption, is determined, without pre-installed instruments, based on the degree of charcoaling of trees affected by the ash cloud. Temperature estimations were performed using Reflectance analysis and microtomography images processing of pine wood charred fragments. The combination of these two independent and well-established methods to organic matter charred in a volcanic environment constitutes a pioneering attempt for the indirect temperature estimation of dilute pyroclastic density currents (PDCs). Charcoal fragments were sampled at different heights along tree trunks outstanding from the PDC deposit. Both the temperatures obtained from charcoal analyses (reflectance and microtomography) and observation of damages to the tree trunks allowed to distinguish: (i) a lower Zone A, which extends 150–180 cm above the top of the PDC deposit, where trunks show peeled bark and multiple lithic impacts; temperature values are equal or slightly higher than the underlying deposit for the entire length of the valley; (ii) an upper Zone B, developed above 150–180 cm from the top of the PDC deposit, where trees are only burned without any block impact marks; temperature estimations for Zone B are comparable with the PDC deposit temperature range from proximal to distal areas. The temperature data indicate that the 11 July, 2015 Colima PDC event, the ash cloud was always thermally coupled with the under-running concentrated flow for the entire length of the ravine, explaining the observed strong vertical uplift of the ash cloud and the substantial absence of ash cloud detachments along flow. A corollary of our study is that, should a detachment have occurred, the ash cloud surge would have had initial temperatures as high as the one carried by the high concentration part of the PDC. A major outcome of our study is that the temperature estimation of ash clouds bears important implication in terms of hazard assessment for pyroclastic density currents along narrow valleys that usually cut the steep slopes of stratovolcanoes.

## Introduction

Pyroclastic density currents (PDCs) generated during dome collapse, also referred to as block-and-ash flows, are among the most hazardous of all volcanic processes in terms of potential damages within their areal extent due to their concentration and velocity (dynamic pressure) and temperature. Many are the cases of fatalities provoked by hot gas and ash mixture flows: i.e. Mount Pelée volcano 1902 eruption, that caused the complete destruction of St. Pierre (Martinique, Antilles), killing 30,000 inhabitants by suffocation and burns^1–3^, El Chichón volcano 1982 eruption (Mexico), during which pyroclastic flow and surges killed more than 2000 people^4^; the 25^th^ of June 1997 eruption Soufrière Hills volcano (Montserrat, Antilles), where pyroclastic surges killed 19 people^5^; Mount Unzen volcano 1991 eruption (Japan), that caused the death of 43 people^6^; the recent 2018 Fuego Volcano eruption (Guatemala), during which 178 people were killed and more than 250 are still missing^7^ and the 2010 Merapi Volcano eruption where more than 200 people died^8–10^.

PDCs are density-stratified flows, constituted by a dense granular basal flow, and an over-riding dilute ash cloud (e.g.)^11–13^. The term block-and-ash flow (BAF) is also here used to refer to the entire PDC generated from the dome collapse, encompassing both the concentrated and dilute portion of the PDC. Most studies on BAF concentrate on the understanding of the basal dense granular flow^14–16^, rarer are on the dilute ash clouds^17–19^, and even less attempted the understanding of the interplay between the basal dense granular flow and the dilute ash cloud^17,20–22^ (and reference therein). Ash cloud surges over-riding the basal dense granular flow can detach and surmount topographic barriers and reach farther distances, constituting in this way a hazard for larger areas than those affected by the basal flow^1,14,19,23–25^, Ash cloud formation has been addressed in literature as the result of different processes as elutriation of the finest particles from a basal dense PDC, ingestion of ambient air and generation of eddies as a consequence of air heated expansion, particles fracturing and as result of explosive dome decompression^22^ (and references therein). Despite our knowledge on the stratigraphical and sedimentological characteristics of ash cloud deposit^26–28^ and the attempts of modelling its flow behaviour in relation with the under-riding basal concentrated granular flow, our understanding of these complex volcanic processes is still far from complete. Physical properties variation such as velocity, density and temperature and how they interact among each other and with the topography during flow is still matter of debate and study. Emplacement temperature of PDCs deposits (principally ignimbrites and BAF deposits) have been determined mostly using paleomagnetic analysis (pTRM^29–40^, (and reference therein) and recently also by Reflectance analysis (Ro%) of charred wood embedded within the pyroclastic deposits^41–46^.

However, a lot still needs to be done to characterize the temperature of the turbulent, dilute portion over-riding the dense basal flows. Very few studies attempted the estimation of ash cloud temperature; at Soufriere Hill Volcano^19^ the ash cloud flow deposits were measured few days after the emplacement using a thermocouple recording very high values (350–410 °C). Direct measurements^47^ of turbulent ash flow generated during the same event (1996–1997) using pre-installed industrial temperature patches reported temperature from 99 °C to 250 °C. In the case of the 2010 Merapi Volcano eruption^9,48,49^ and Tungurahua Volcano^50^ researchers constrained dilute PDC cloud temperatures using wood combustion values, melted plastic pots, nylon clothing and thermal effects on building components (100–300 °C and 100–250 °C respectively).

In this work, we characterise the temperature of the ash cloud associated with the 11^th^ of July 2015 block-and-ash flow at Colima, by studying the charring intensity of trees affected by the ash cloud. Through the application of two independent proxies as Reflectance analysis and micro-tomography images processing of charred trees, we reconstruct the temperature variation from proximal to distal areas, and also in vertical where standing trees could be sampled at different heights. By comparing the temperature data of the ash cloud presented in this study, with the temperatures of emplacement of the basal granular flow deposits^32^ an interpretation is possible about the degree of thermal coupling between the two portions of the PDC.

This study represents the first endeavour of ash cloud indirect temperature evaluation without pre-installed instruments^47^ and it constitutes an advance in charcoal optical analysis validation as a temperature proxy in a volcanic environment^9,32,42,43,45,46^. The results obtained in this study greatly contribute to improve the volcanic hazard assessment in areas densely inhabited, that are usually not only directed involved by the emplacement of dense block-and-ash flow, but also affected by the diluted over-riding ash clouds. Furthermore, considering the similarities (valleys morphology, volcanic activity type and vulnerability) of Volcán de Colima with Fuego Volcano in Guatemala^51^, the hypothesis that a similar scenario as the 2018 Fuego volcano eruption may occur is very likely in the specific case of Volcán de Colima. Of the great number of fatalities (178 people had been killed and nearly 250 are still missing^7^) and injuries occurred at San Miguel Los Lotes and Escuintla towns during the Fuego Volcano 2018 eruption, many occurred in the areas affected by the dilute parts of the current, where severe burnings clearly demonstrate the hazard posed by their temperature.

## Volcanological setting

**Figure 1:**
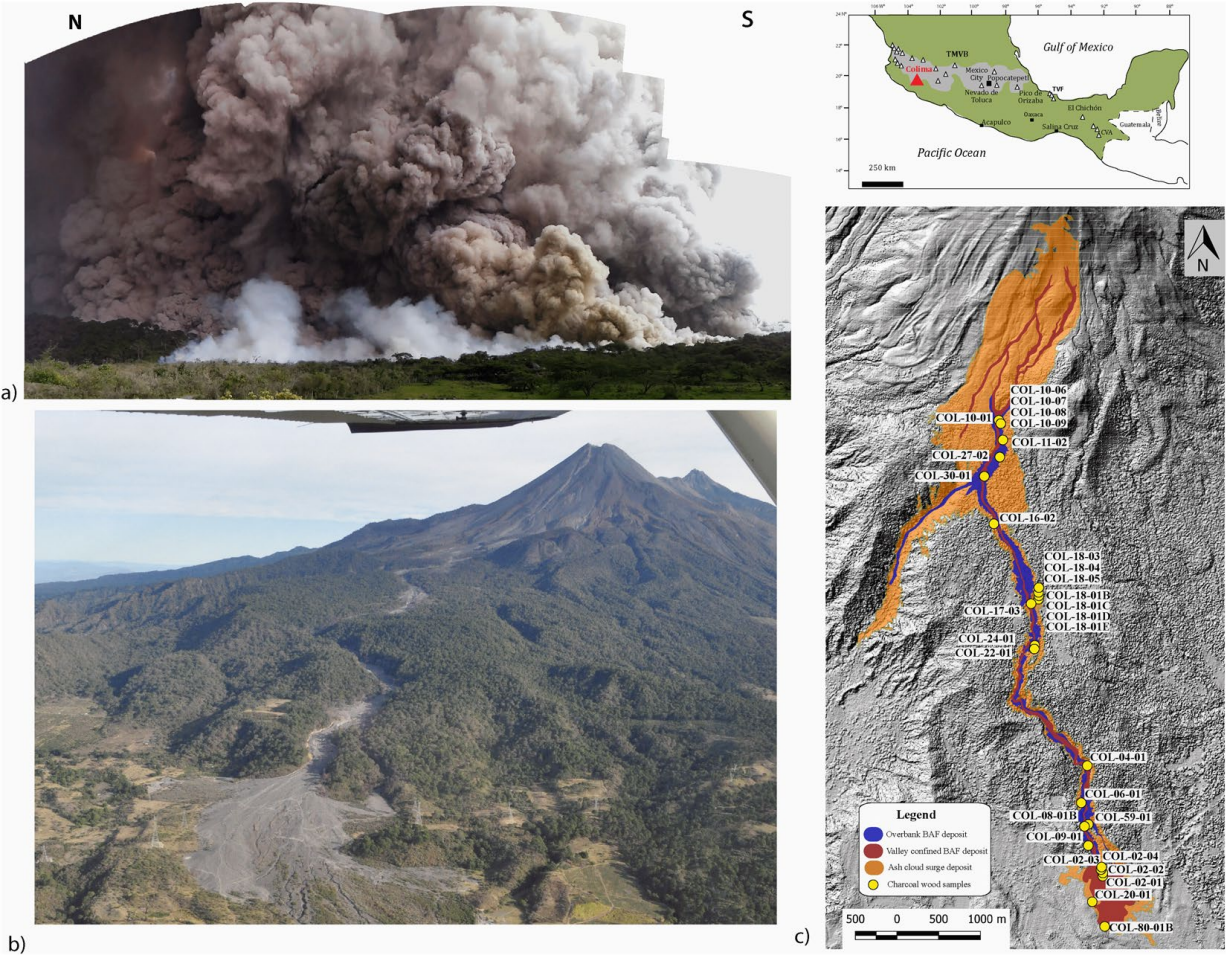
(**a**) Collage of photos taken during the 11^th^ of July of the BAF generated by the dome collapse of Volcán de Colima. (**b**) Aerial photo of the Montegrande ravine after the 2015 event. The BAF deposit filled the narrow valley destroying and charring trees, reaching the end of the ravine at 10.5 km from the vent. (**c**) Map of the charcoal fragments sampling locations along the Montegrande ravine.

Volcán de Colima is part of the Quaternary Trans-Mexican Volcanic Belt (TMVB, Fig. 1) and it is located on its western margin. Volcán de Colima constitutes one of the most active volcanoes in Mexico and its activity has been mainly characterised by cycles of vulcanian eruptions associated to summit dome growth and subsequent collapse with the emplacement of block-and-ash flow (BAF) deposits along main ravines^52,53^ (and reference therein) culminating sometimes in Plinian and sub-Plinian eruptions (i.e. the 1576, the 1818 and the 1913 eruptions^54,55^).

## The July 2015 eruption

Following a period (2013–2014) of intense vulcanian explosions on September 2014 a new dome rapidly grown in the summit of the volcano. At the beginning of 2015 Volcán de Colima produced several gas-and-ash plumes per day rising to altitudes of 5.5–7.3 km. On the 10^th^ of July part of the dome and of the old crater collapsed along the SSW flank and generating BAFs that filled the main channel along the Montegrande ravine, up to a distance of ≃9 km from the vent^52,53,56^. This event lasted for 52 minutes, no eruptive column was associated to the partial dome collapse, but the phoenix plume from the BAFs reached ≃4 km in height and dispersed up to 150 km towards SW where ash fall was reported^52,53,56^. The day after, the 11^th^ of July, the remaining dome and new material rapidly extruded from the conduit collapsed along the same ravine. This event, also not associated to eruptive column dynamics, lasted for almost two hours, resulted in a series of BAF deposits with vesiculated lava fragments, and distributed as lobes along the Montegrande ravine^56^. These BAFs topped the previous deposit, over-spilled from the Montegrande ravine up to its distal reach where it opens to a fan-like shape at ≃10.5 km from the vent (Fig. 1b). The total volume estimated ranges from 7.7 × 10^6^ m^3 ^^56^ (including fall-out ash)^53^.

## Thermal characteristics of the BAF deposits from previous study

Thermal remanence magnetization (TRM) analysis of lithic clasts and charcoal Reflectance analysis of charred wood fragments, both embedded within the 2015 BAF, revealed for the concentrated BAF deposit emplacement temperatures varying from 345–385 °C in valley-confined area (from 3.5 to 8.5 km from the vent) and ≃170–220 °C (from 8.0 to 10.5 km from the vent) in unconfined distal area^32^. Over-bank and valley temperature results did not display significant differences.

The authors highlighted that, despite the long run-out (≃10.5 km), the BAF maintained high temperature till the end of the valley. The higher temperature values were found in the central sector of the ravine, where the channel is narrower and deeper than in proximal and distal valley sectors.

This difference in temperature emplacement along the Montegrande ravine was attributed to the combined relationship between depositional and transport processes and topography confinement.

## Results

### The PDCs impact on the landscape

The 2015 dome collapse event represents one of the largest and farthest-reaching block-and-ash flow events at Volcán de Colima. While the dense basal avalanche of the PDCs remained mostly topographically confined within the Montegrande ravine (Fig. 1a,b)^32,52,56,57^, the over-riding dilute ash cloud covered a wider area especially in the proximal sector (Fig. 1c), where the break in slope (from 45° to 15° in less than 1800 m) is considerable, and in the distal, unconfined area where it expanded laterally. Despite its dilute nature, the lateral expansion of the ash cloud resulted however limited, not exceeding 150 m away from the centre of the valley^56^, following the same path of the underlying concentrated flow.

**Figure 2:**
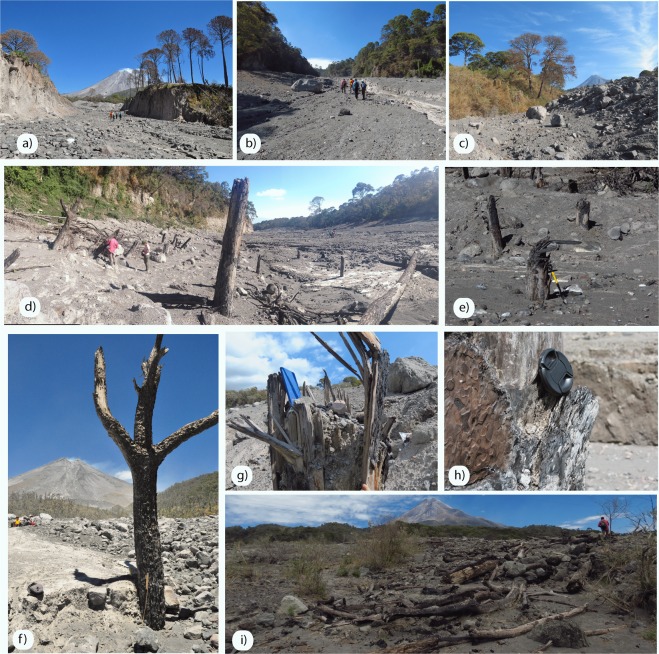
Images captured along the Montegrande ravine of trees buried and charred by the BAF on the over-banks and in the valley centre in proximal area (**a**–**c**), central area (**d**–**h**) and distal area (**i**).

As displayed in Fig. 1c,d, the Montegrande ravine is extremely vegetated by pine trees (e.g. Pinus harwegii) and spruce trees (e.g., Abies guatemalensis and Abies Jaliscana^58^). The July 11th 2015 PDCs destroyed completely the vegetation within the valley by uprooting, breaking, burying and carbonizing trees higher than 30 m (Fig. 2a–d). The still standing trees in the valley centre have their bases immersed in the deposit and are intensely damaged by the flow passage.

**Figure 3:**
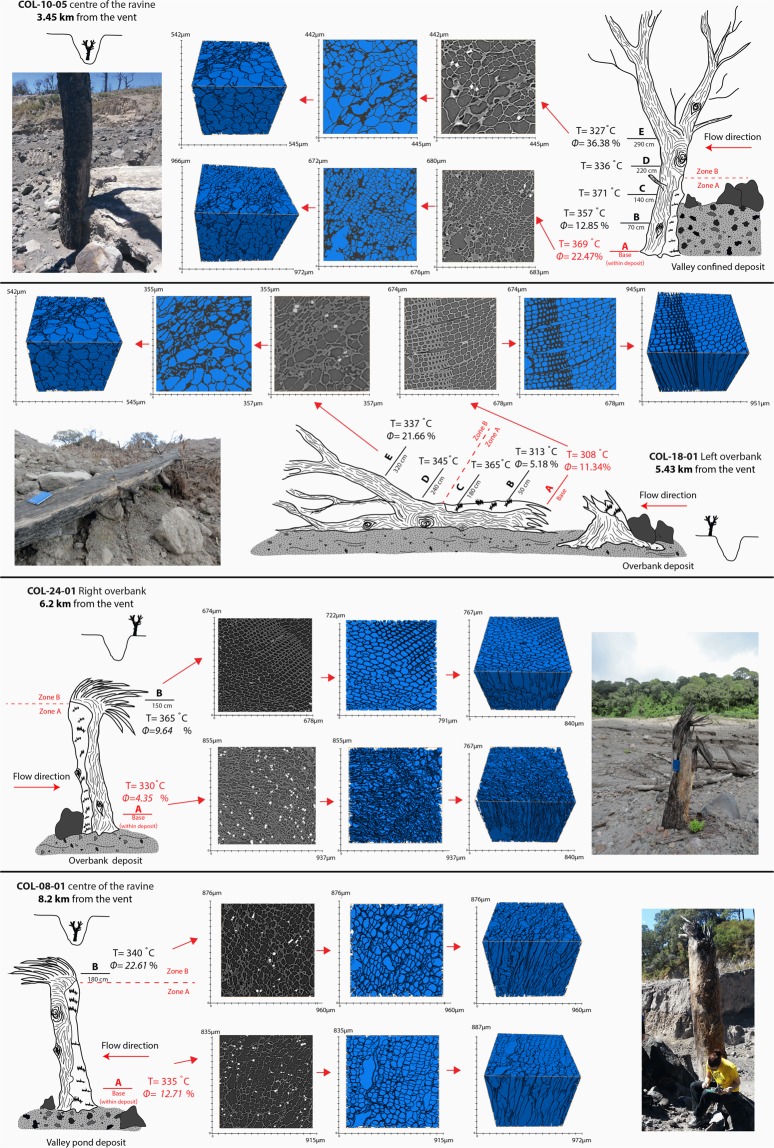
Combination of Ro% and Micro-tomography (ɸ) analyses carried on four representative charred trees located in proximal (**a**) central (**b**,**c**) and distal (**d**) areas. Micro-porosity values indicate variation respect to raw wood. Charring intensification, and therefore Reflectance degree, results parallel to increase in cell-walls diameter due to pyrolysis process.

Despite their size and resistance as adult pine trees, all of them lost the canopy, are broken in half with the bark abraded along the upstream side (Fig. 2e–h). The remaining tree portion result charred, with a burn extension involving only few millimetres (<3 mm) of the tree surface leaving the wood inside unaffected (Fig. 2h). This suggests an intense but short-lived heat exposure. In addition, the upstream trees side displays multiple lithic clasts impacts that are clearly visible from the deposit surface level up to 150–180 cm in height along the trunk (Fig. 2f). This characteristic is recognisable in all the analysed trees from proximal to distal areas. Above this height (180–150 cm) still standing trees in the valley centre did not display signs of lithic impacts; the bark is not removed but only burned till the top of the remaining tree (Fig. 3). The over-spilled flow left on the valley sides several trees broken in half and bent in the flow direction (Fig. 2a); the still standing trees, resulted burned, completely naked as their leaves were totally blown away from the branches. Few meters distant from the valley edges trees are only partially affected by the flow; their canopy is half burned and half still green. The trunks are superficially (<3 mm) burned along the side facing the flow but the lithic marks are less severe than in the valley centre, involving the first 50–150 cm of the trunk from the deposit level.

Numerous are the trees totally uprooted by the flow and deposited at the end and along the sides of the valley (Fig. 2i).

### Reflectance analysis on charred wood

Twenty-eight wood fragments were selected and sampled for the estimation of the ash cloud temperatures. The trees location is reported in Fig. 1d; they were chosen along the total length of the Montegrande ravine, both in centre of the valley and along the over-banks to better asses the temperatures variation laterally and from proximal to distal areas. All the trees selected were *in situ* and almost the totality of them are pine trees (e.g. Pinus harwegii) and few spruce tree (e.g., Abies guatemalensis and Abies Jaliscana^58^). On the contrary charred trees totally uprooted and emplaced on the surface of the deposit were discarded because of their unknown original location. The sampling strategy focused on collecting wood fragments from deposit surface to 150–180 cm in height and from the above tree portion simply burned.

**Figure 4:**
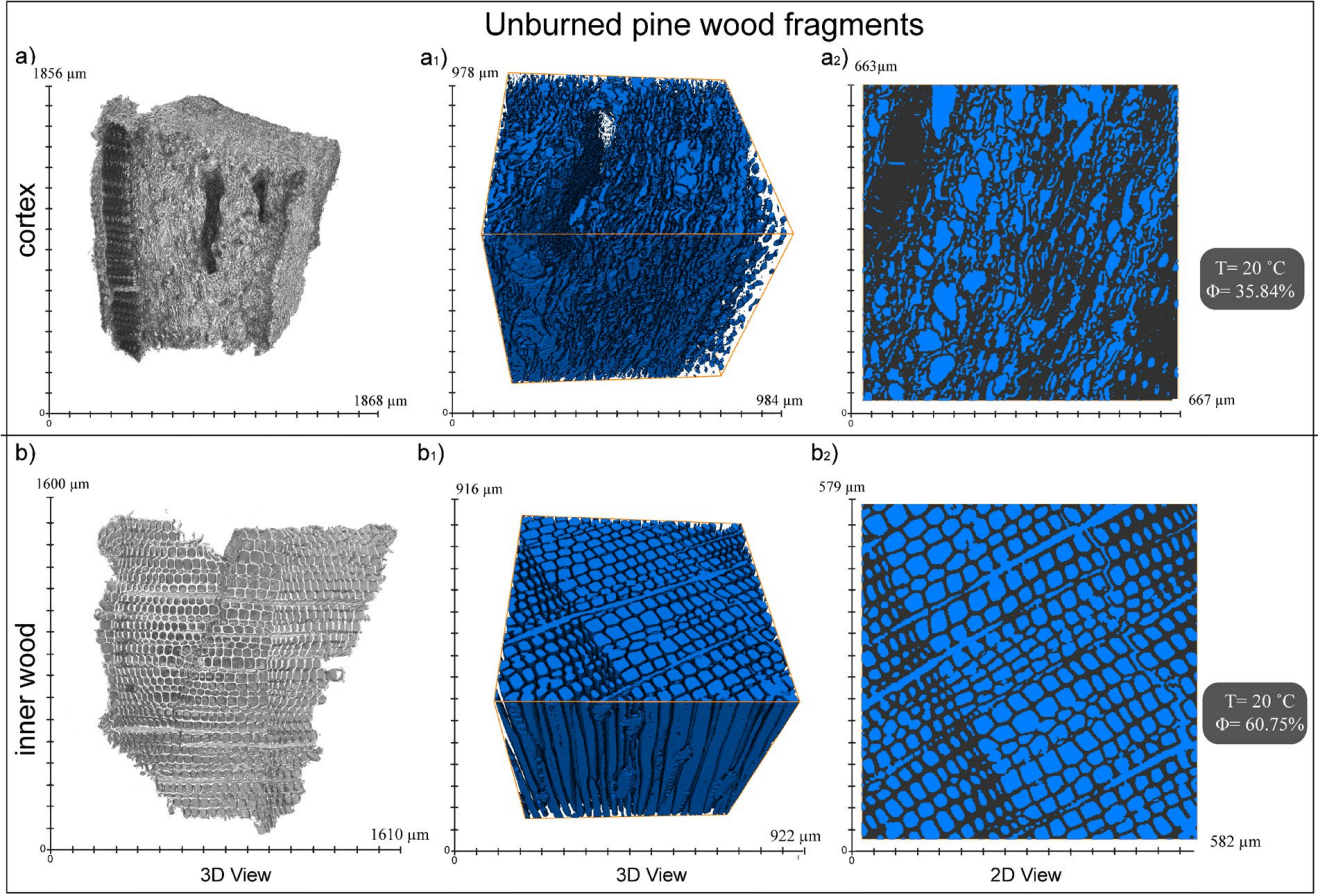
3D and 2D images of micro-porosity analysis of raw pine outer bark (**a**) cortex: irregular structure with elongated, wavy cells and sapwood (**b**) inner part: regular structure with well-organized rounded cells.

Optical analysis of the 28 selected trees inferred Reflectance percentage ranging between 0.253 ± 0.021 and 1.768 ± 0.068, that correspond to a variation in temperature between 306 °C and 464 °C respectively according to^41^ pine wood pyrolysis curve^32^ (Table 1). Under reflected light and oil microscope, charcoal fragments appear well visible with different morphologies, cell shape and colours ranging from yellow-gold to olive green according to their reflectivity. Bark (cortex) samples display wavy texture with elongated and irregular cell walls and presence of orange resin. In these samples, high and low reflective fragments with different colour are present contemporaneously. Sapwood (inner part) samples is characterised by a more regular “beehive” structure, which cells are round to sub round, well distinguishable (Figs 3 and 4) and yellow in colour.

**Table 1:** Data summary of the charcoal fragments sampled for the Reflectance analysis and temperature evaluation.

Location	Fragment name	Latitude	Longitude	Sampling zone along trunk	T °C zone	Ro% average	standard deviation	T °C^41^	Error (based on st.dev.)
Valley conf	COL-10-03	644410	2154898		BAF deposit	0.554	0.028	345	
	COL-10-01			200 cm	Zone B	0.676	0.033	354	+4.0/−3.3
Valley conf.	COL-10-05	644414	2154885		BAF deposit	0.798	0.035	369	
	COL-10-06			70 cm	Zone A	0.698	0.034	357	+2.9/−3.4
	COL-10-07			170 cm	Zone A	0.83	0.035	371	+3.6/−3.2
	COL-10-08			220 cm	Zone B	0.509	0.028	336	+3.5/−4.0
	COL-10-09			290 cm	Zone B	0.433	0.03	327	+3.8/−3.7
Over-bank	COL-11-03	644445	2154691		BAF deposit	0.429	0.024	332	
	COL-11-02			100 cm	Zone B	0.698	0.032	357	+3.2/−3.8
Valley conf.	COL-27-01	644409	2154489		BAF deposit	0.863	0.054	376	
	COL-27-02			150 cm	Zone B	0.592	0.049	345	+5.5/−5.4
Valley conf.	COL-30-01	644227	2154141	150 cm	Zone A	1.385	0.071	428	+6.8/−7.1
Over-bank	COL-16-04	644339	2153689		BAF deposit	0.632	0.051	353	
	COL-16-02			50 cm	Zone A	1.339	0.067	423	+7.0/−6.2
Over-bank	COL-18-01A	644878	2152872		BAF deposit	0.197	0.019	308	
	COL-18-01B				BAF deposit	0.242	0.063	313	
	COL-18-01C			50 cm	Zone A	0.776	0.03	365	+3.2/−3.9
	COL-18-01D			180 cm	Zone B	0.588	0.032	345	+3.6/−3.6
	COL-18-01E			240 cm	Zone B	0.52	0.032	337	+3.5/−3.0
Over-bank	COL-18-03	644867	2152908	50 cm	Zone A	0.992	0.051	388	+5.5/−5.2
	COL-18-04	644880	2152877	50 cm	Zone A	1.187	0.043	408	+4.6/−4.2
	COL-18-05	644868	2152848	50 cm	Zone A	0.899	0.047	378	+5.3/−4.6
Over-bank	COL-17-01	644773	2152779		BAF deposit	0.514	0.03	341	
	COL-17-03			150 cm	Zone A	1.768	0.068	464	+6.4/−6.0
Over-bank	COL-22-02	644821	2152258		BAF deposit	0.371	0.028	326	
	COL-22-01			150 cm	Zone A	1.125	0.054	402	+5.4/−5.6
Over-bank	COL-24-02	644816	2152259		BAF deposit	0.408	0.022	330	
	COL-24-01			150 cm	Zone A	0.776	0.035	365	+4.0/−3.6
Valley conf.	COL-04-02	645441	2150807		BAF deposit	0.416	0.024	325	
	COL-04-01A			100 cm	Zone A	1.483	0.08	437	+8.1/−7.3
Valley conf.	COL-08-01A	645415	2150085		BAF deposit	0.454	0.026	335	
	COL-08-01B			180 cm	Zone B	0.54	0.036	340	+4.3/−3.8
Valley conf.	COL-59-01A	645445	2150097		BAF deposit	0.28	0.018	317	
	COL-59-01D			8 m	Zone B	0.392	0.023	323	+2.2/−3.1
Over-bank	COL-09-02	645434	2149839		BAF deposit	0.486	0.029	338	
	COL-09-01			80 cm	Zone A	1.372	0.059	426	+6.4/−5.2
Distal fan	COL-02-01	645636	2149535	170 cm	Zone B	0.461	0.034	330	+4.2/−3.5
	COL-02-02	645637	2149568	170 cm	Zone B	0.356	0.027	318	+3.5/−2.7
	COL-02-03			120 cm	Zone A	0.385	0.022	322	+2.2/−2.8
	COL-02-04	645617	2149595	120 cm	Zone A	0.408	0.027	324	+3.4/−2.7
	COL-20-01	645510	2149153	170 cm	Zone B	0.253	0.021	306	+2.9/−2.0
	COL-80-01A	645647	2148871		BAF deposit	0.488	0.026	338	
	COL-80-01B			150 cm	Zone A	0.784	0.032	366	+3.5/−3.4

Site location along the valley, position within the BAF, Lat (latitude), Long (longitude), Ro% mean and standard deviation are reported. Reflectance data conversion into emplacement temperature values (and relative errors based on standard deviation) are listed according to 3 curve (See)^32,41^.

Temperature values were distinguished between data relative to wood fragments collected within the first 150–180 cm above the actual BAF deposit surface (here there in called Zone A), and results obtained from charred wood samples collected above 150–180 cm (here there in called Zone B).

Reflectance analysis of the selected fragments sampled from valley confined pine trees infer temperatures relative to Zone A that vary from 357 °C to 426 °C (Table 1); and temperatures between 323 °C to 354 °C for Zone B. On the overbanks, the temperature evaluation indicates values from 365 °C to 464 °C within Zone A, and temperatures between 337 °C and 357 °C within Zone B. Proceeding towards the distal fan the temperature values inferred by Ro% analysis decrease along the final confined sector of Montegrande ravine, varying between 322 °C and 366 °C in Zone A, and between 306 °C and 330 °C within Zone B (Table 1).

### Micro-tomography analysis

A total of 10 fragments (See Table 2 and Fig. 3), were selected among the samples composing the data set used the ash cloud temperatures estimation with Ro%. In order to detect variation in physical properties due to pyrolysis^59,60^ for each pine tree were selected fragments from Zone A and Zone B. For pine trees COL-10–05 and COL-18-01 the analysis of intermediate points at different heights from the base was possible (Table 2 and Fig. 3).

**Table 2:** Data results of micro-porosity analysis on charred fragments of four representative pine trees located in proximal area (COL-10-05) central area (COL-18-01 and COL-24-01) and distal COL-08-01).

		Fragment position	Sample	Type wood texture	Micro-porosity	Micro-porosity variation respect to raw wood
Pine tree COL-10-05	Zone A	Base	Col-10-05-A	outer bark	58.31%	+22.5%
	Zone A	70 cm	Col-10-05-B	outer bark	48.69%	+12.9%
	Zone B	200 cm	Col-10-05-D	outer bark	72.22%	+36.4%
Pine tree COL-18-01	Zone A	Base	Col-18-01-A	sapwood	72.09%	+11.3%
	Zone A	50 cm	Col-18-01-B	sapwood	65.93%	+5.2%
	Zone B	200 cm	Col-18-01-D	outer bark	57.50%	+21.7%
Pine tree COL-08-01	Zone A	Base	Col-08-01-A	sapwood	73.47%	+12.71%
	Zone A	180 cm	Col-08-01-B	outer bark	58.45%	+22.61%
Pine tree COL-24-01	Zone A	Base	Col-24-02 B	outer bark	40.19%	+4.35%
	Zone A	150 cm	Col-24-01 A	sapwood	70.40%	+9.64%
Pine tree COL-81-01		Raw wood	Col 81-01-A	outer bark	35.84%	/
		Raw wood	Col 81-01-B	sapwood	60.75%	/

Micro-porosity analysis of un-burned cortex and sapwood pine fragments (COL-81-01) in order to valuate porosity variation of burned samples from raw samples.

Micro-tomography image analysis of the representative charred fragments displayed a noticeable thinning of the cell walls of wood fragments from the base of the tree to the top (Fig. 3). The collapse of wood texture resulted in an increase in cell size, due to the coalescence of several cells, and consequently to the growth of pore connectivity (micro-porosity).

As reported in Table 2 and Fig. 4, micro-porosity of carbonised fragments is higher respect to raw pine values. The variation was estimated by comparing the carbonised fragment with the corresponding raw sample (outer bark or sapwood). The correlation between raw and charred fragments revealed that micro-porosity increases upwards from the deposit level, regardless of the type of fragment (bark or sapwood. Figures 3 and 4 and Table 2).

## Discussions

Charcoal Reflectance and wood micro-porosity analyses together with field evidences highlight the presence of two distinct Zones: A and B. One relative to the first 150–180 cm of trunks/trees from deposit surface, intensely damaged by blocks impacts, and a second one related to the above charred tree portion, free of lithic clasts marks. Temperatures that affected Zone A are from 15 to 107 °C (average 55 °C) higher to the upper part Zone B (Table 1), and from 15 to 90 °C (average 50 °C) respect to the corresponding BAF deposit estimated in^32^. The presence of two distinct temperature zones was detected both on the over-banks (365–464 °C Zone A and 337–357 °C Zone B) and in valley confined area (357–426 °C Zone A and 323–354 °C Zone B). Along the distal fan the range of temperatures relative to the two trunk zones are comparable (322–366 °C Zone A and 306–330 °C Zone B) (Table 1).

This temperature variation from deposit surface to trees’ top, is corroborated by wood texture micro-tomography data that display an increase in wood pore connectivity parallel to intensification of Reflectance degree. According to pyrolysis experiments on pine wood^60,61^, increase in micro-porosity are wood physical properties changes attributable to increase in temperature.

Micro-porosity analysis of the four selected trees (Fig. 3), located along the Montagrande ravine displays the maintenance of such increment in temperature upwards, at least up to a distance of 8.2 km (COL-08-01 tree; Fig. 3) from the vent.

### The block and ash dense basal flow temperature and dynamics

**Figure 5:**
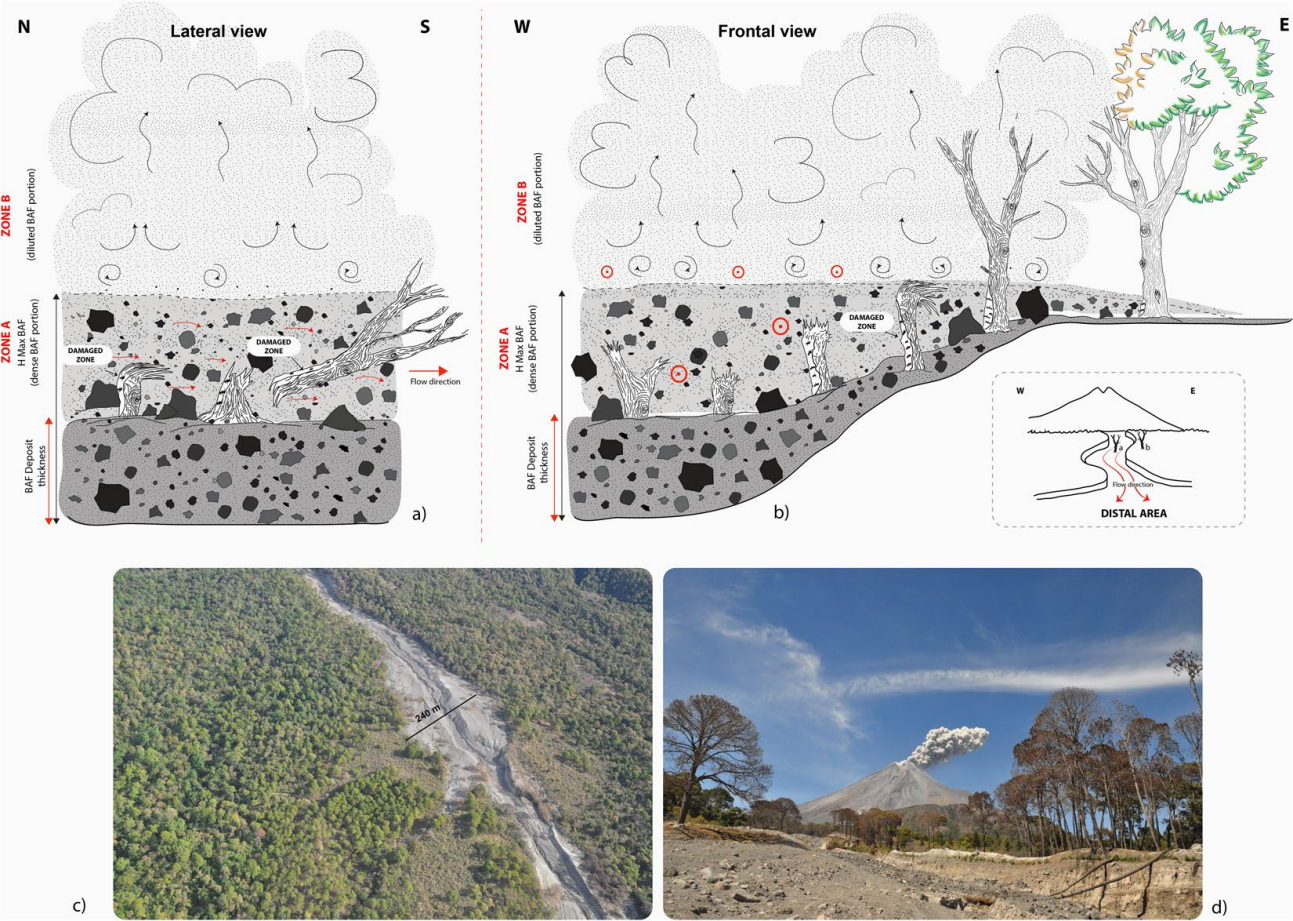
Schematic representations of the Block and ash flow dynamics from lateral view (**a**) and frontal view (**b**). During the event the maximum height reached by the dense part of the flow was 150–180 cm above the actual deposit level. This is testified by the multiple lithic clasts impacts on the still standing trunks. (**c**) Aerial view of the maximum lateral extension of the BAF deposit within the Montegrande ravine, (**d**) image taken in the middle of the ravine towards NE, showing the still standing trees on the overbanks.

Field evidences highlighted that Zone A was directly affected by the concentrated basal flow. The multiple lithic impacts and the comparable extension of trunk damage area and the bark peeling displayed by all the trees, testify that the granular basal flow was at least 150–180 cm thicker (within the valley) than the resulting deposit after eruption. Instead the absence of lithic clasts marks above 150–180 cm in height indicates that the upper part of the trees (Zone B), was never in contact with the dense basal flow, but only with the most diluted flow portion (Fig. 5).

This evidence has great importance in terms of timing of charring events, as the charred trees recorded not only the temperature dissimilarity within and between the concentrated basal portion and the dilute ash cloud, but also the possible temperature and thickness fluctuations during the flow event duration. Due to its not retrograde nature, the process of carbonification has recorded over time only the maximum temperatures experienced by the tree trunks during the flow transit at different heights.

This allows us to reconstruct the thermal and dynamic of the dense basal flow history at different steps. The high temperature reported by charcoal Reflectance analysis in Zone A (Table 1) was recorded during the passage of one or multiple pulses. This implies that emplacement temperatures estimated within the deposit at any location *(cf*.)^32^, may not correspond to values estimated from *in situ* (i.e. rooted) trees in Zone A, as Zone A of standing trees records the maximum temperature occurred during flow, while temperatures of the deposit refer to the diachronous event of deposition, which may occur any time later (in case of backstepping of deposition) or earlier (in case of forestepping).

**Figure 6:**
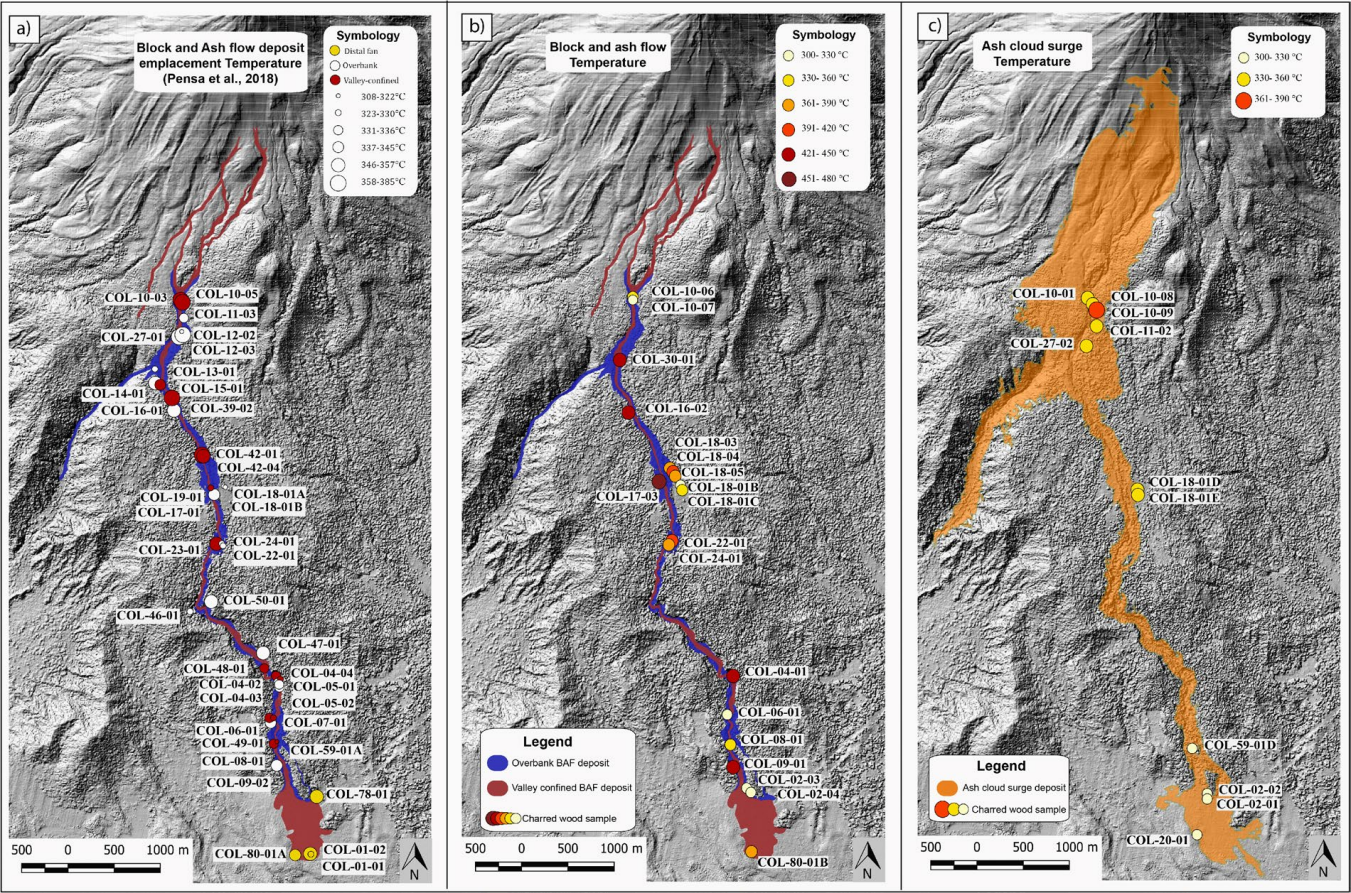
Maps of temperature variation from proximal to distal areas of the (**a**) Block and ash flow deposit (modified after^32^); under-running concentrated flow portion (Zone A) (**b**), and of the over-riding ash cloud (Zone B) (**c**).

Figure 6a,b shows respectively the BAF deposit temperature map (modified after^32^) and the temperature estimations retrieved in Zone A. Our data show the temperature which affected trees within Zone A were 15–90 °C higher than the deposit emplacement temperature^32^ for the entire length of the Montegrande ravine (Fig. 6a,b). This implies that incorporation of substrate materials (bulking process), vegetation, together with gasses loss could have affected the temperature during the deposition process, accounting for a maximum of 90 °C compared to flow temperature.

Trees on the over-banks, although with smaller extension of the charred zone, experienced similar differences in temperature between the deposit (220–260 °C^32^) and the Zone A (365–464 °C), where temperatures are comparable to the valley confined results (Zone A valley confined area 357–426 °C Fig. 5b).

At the distal fan, BAF deposit temperature values and those of Zone A are perfectly comparable: 325–357 °C within the deposit (See^32^), and 322–366 °C in Zone A, suggesting that at distal locations the BAF was fully depositional.

### The over-riding diluted ash cloud temperature and flow dynamics

Charred wood fragments collected above 150–180 cm (Zone B) (Figs 3 and 5) never came in contact with the basal granular flow, but only with the most diluted portion of the BAF. Temperature values indicate that despite the slightly lower temperature ranges (337–357 °C on the over-banks and 323–354 °C in valley confined area) respect with the under-running concentrated flow (15–107 °C lower, average 55 °C), the over-running diluted ash cloud maintained high temperature values along the entire length of the Montegrande ravine, decreasing only in correspondence of the valley opening (306–330 °C) (Fig. 6c).

The ash cloud temperature values can be interpreted by considering the relative role of air entrainment, the degree of fragmentation, mass heat flux from the underlying dense granular flow, particles depositional dynamics and morphology of the channel^22^. Several numerical models^62–64^ (reference therein) and physical experiments^17,20^ (and reference therein) proved the strong dependence of maximal run-out distance of an ash cloud with the flow volume, sedimentation rate and the mass of air ingested during the flow (see^38^). The development of convective ash cloud as a consequence of fine particle elutriation from the dense basal flow, depends mainly by the mass of air entrainment^20,62^ and also by the thermal state and kinetic energy of the pyroclastic density currents^17^. The ability of ingesting great quantity of air depends on the relationship between the potential energy necessary to enter in the upper portion of the flow and the kinetic energy of the same flow that rules the entrainment^18,62^. As a consequence of the conspicuous ingestion of air, the density of the flow decreases rapidly, resulting in short run-out distances and deposit emplacement cold temperatures.

As stated by^17^ the lift-off of the dilute ash cloud above the basal dense granular flow depends on flow temperature. Experiments carried on air entrainment and run out distances of dilute pyroclastic density currents demonstrated that heated ash flows generate lift-off plumes in early stage of the flow compared to non-heated flows. While the latter continue to propagate generating at the head of the flow an uplift plume in late stage of the experiment and with a radial dispersion, heated ash currents display narrow dispersal pattern. The tighter spreading has to be attributed to an earlier lift-off of the current. The formation of the ash buoyancy conveys an upward-direction that limits the air entrainment from the flow front and the upper margins, enhancing air ingestion from the lateral boundaries, preventing concurrently the lateral spreading^17,18^.

The BAF generated during the 2015 Volcán de Colima eruption, reached 10.5 km in distance from the vent, over-filling the Montegrande ravine and displaying a limited lateral spreading of 150 m from the centre of the valley^56^. As reported in^32^ the emplacement temperature of the BAF deposits remained high, 345–385 °C, as long as the deposit was confined within the valley; dropping at ≃170–220 °C in unconfined distal area. Field evidences and videos recorded by witnesses during the eruption, revealed that the over-riding ash cloud developed from the early stages of the dome collapse, expanding meanly upwards (4 km in height^52,56^), remaining un-detached from the under-running concentrated flow for its entire length. Despite the sinuous trend of Montegrande ravine, the dilute ash flow did not separate from the basal granular flow, except in proximal area, where it detached over-spilling in the surrounding area, due to the abrupt change in slope (from 45° to 15°) at the base of the edifice and in distal area where it spreads radially. Similar behaviour of over-riding ash cloud and basal block-and-ash flow detachment due to topographic change in slope or valley direction was recognised during the 1982 El Chichon Volcano, Mexico^65^; the 1991 eruption of Unzen Volcano^6^ and the 2010 eruption of Merapi Volcano, Indonesia^6,8,9^.

The temperature estimations of the over-riding ash cloud obtained in this study indicate high values both in valley confined and in over-banks areas. In particular, reflectance data from charcoal fragments sampled within Zone B, do not identify any thermal vertical gradient, indicating that the ash cloud temperature remained constant upwards (See Table 1) at least up to two meters from the boundary with Zone A.

As expected, the ash cloud surge temperature decreases slightly with the increase in the distance from the vent but remaining almost equivalent to those of the underlying deposit along the entire length of the valley. The maintenance of such a high temperature until the end of the valley confinement is corroborated by the minor drop in temperature (323–354 °C valley confined; 306–330 °C distal fan) showed by the ash cloud along the distal fan. This could mean a very low ingestion of cold air within the basal dense flow from frontal and upper margins; by contrast the lateral entrainment of air within the ash cloud was enhanced so much to inhibit the lateral expansion of the ash flow as described by^17^. This hypothesis is confirmed by the presence of still standing pine trees along the edges of the Montegrande ravine, with canopy half green facing out the channel, and half discoloured by the hot gases of the ash cloud (Fig. 5). This strong thermal lateral confinement was also identified at Mt, Unzen during the 1991 dome collapse, when the ash cloud remained confined, ascending convectively without burning the trees along the valley edges.

### Relationship between BAF and ash cloud

The temperature data obtained from Zones A and B highlight the inter-relationship that exists between the basal dense and the diluted flows when decoupling does not take place. Despite the numerous examples in literature of decoupling of dilute, turbulent ash cloud surges from dense basal flows (Mt Pele´, Martinique in 1902; Unzen, Japan 1991; Soufriere Hills Volcano, Montserrat 1997 and Merapi Volcano, Indonesia 2010) at Volcán de Colima the separation did not take place efficiently. Although meandering, the narrow topographic conditions of the Montegrande ravine and the main vertical expansion of the ash cloud promoted the conservation of high temperatures of both flows (at least at the base of the ash cloud). This implies that the ash cloud was constantly thermally powered by the under-running concentrated flow for the entire length of the ravine.

The maintenance of such interdependence is validated by the similarity of temperatures experienced by trees on the over-banks (few metres from the valley edges) to that ones in valley centre. Such strong thermal coupling between the dense and diluted flow is also proved by the contemporaneous slight temperature decrease with distance from the vent (Fig. 6b,c). These results are very important in terms of hazard as the conservation of high temperature at noticeable distances from the vent (i.e. 8 km at Montegrande ravine) constitutes a potential risk for communities located nearby. In addition of being possibly reached by the flow dense basal portion, the nearby villages (as La Yerbabuena and Queseria in Colima), can be subject to hot ash cloud surge impact in case of detachment.

The point where the ash cloud detachment may occur is of extreme importance. Usually the first point in which a separation from the basal flow takes place is the proximal area, due to the strong break in slope at the base of the edifice (45–15 degrees at Volcàn de Colima^56^) Here temperature of dense and diluted flows decreases substantially due to the current spreading and the enhanced air entrainment^9,32^.

Ash cloud can also decouple from the basal flow due to abrupt change in topography further downstream. As it happened in 1991 at Mt. Unzen Volcano and at Soufriere Hill Volcano in 1997, at sharp valley bends, the ash cloud detached and propagated like a surge separately from the main flow^22^. Due to their relevant lateral motion component the diluted ash cloud surges can spread easily and quickly affecting a larger area than the topographic controlled dense basal flows. In the case of the 1991 Mt. Unzen event, the detachment of the ash cloud killed 43 people essentially for the thermal impact^6^. It is therefore evident that in case of strong topographic confinement, maintenance of high temperature and inefficient decoupling between the diluted and the concentrated flows (as for Volcán de Colima), a possible ash cloud detachment al medium or high distances from the vent, at similar temperature conditions of the parental dense flow, can have major deadly consequences compared to cold ash cloud surges. In the case of Volcán de Colima this means that from 4 to 9 km from the vent an ash cloud surge of ≃300–350 °C can detach and spread laterally.

## Conclusions

The aim of this study is to reconstruct the ash cloud flow temperature and flow dynamics by describing the effects on the landscape after its passage. Through field evidences, temperature estimations by using Reflectance and Micro-tomography analyses of charred wood we constrained the ash cloud flow process and its relationship with the under-running dense flow and topography confinement. Temperature data of charred trees directly affected during the 2015 eruption revealed the presence of two different temperature zones (Zone A, and Zone B).

The presence of lithic clasts marks and high temperature values indicate that Zone A was directly in contact with the flowing dense part of the BAF that was 150–180 cm higher than the actual deposit surface. Contrarily, the absence of lithic clasts impacts within the upper Zone B infers that this area never came in contact with the dense basal flow, but only with the dilute ash cloud.

Contrarily to many other cases, as El Chichon 1982^65^, Ngauruhoe volcano 1975^66^, Tungurahua volcano 2006^50,67^. Merapi volcano 2010^8–10,12,68^ and Mt Unzen 1991^6^, Soufriere Hills Volcano, Montserrat^19^, the over-running ash cloud did not decouple from the parental dense bulk flow; this resulted in a continue “heat feeding” from below to the dilute flow. The continue heat release contributed to maintain high temperatures along the 10.5 km long Montegrande ravine.

Despite the ravine sinuosity, the ash cloud did not detach from the base but remaining confined within the ravine flanks, further reducing the lateral entrainment of cold air. This is corroborated by the presence of un-burned trees close to the over-bank’s edges.

The results here presented have important implications, since that under particular topographic conditions, ash cloud can maintain a strong bond with the under-running dense flow, maintaining very high temperatures for long distances from the vent. This constitutes a potential hazard in case of ash cloud surge detachment caused by strong direction variation as for sharp valley bends. Such scenario, as happened in Guatemala during the Fuego Volcano 2018 eruption, will imply the formation in medium-distal areas of lateral ash cloud surges at the same high temperature of the parental dense flow. This represent a further risk for the numerous communities located directly at the end of the several valleys along the Volcán de Colima flanks.

## Methods

The burning temperatures to which the sampled trees were subjected were estimated using optical and micro-tomographic analyses on charcoal fragments collected from trees involved in the eruption. Reflectance analysis (Ro%) of charred wood, that correlate the degree of carbonification of wood fragments with heat exposition and duration, burning/burial mode and deposit charring temperature, has been recently validated as excellent proxy for temperature assessment in volcanic environment (i.e. Soufriere Hills Volcano, Montserrat^19^; Taupo Volcano, New Zealand^43^; Vesuvius Volcano, Italy^25,42,69^; Fogo Volcano, Azores^45^; Merapi Volcano, Indonesia^9^; Colima Volcano, Mexico^32^).

Based on South Africa wildfire studies^59,60^ and laboratory experiments on wood physical properties behaviour during pyrolysis^61^, micro-tomography analyses were undertaken on selected raw and charred pine wood fragments in order to visualise and quantify the thermal degradation caused by the BAF and ash cloud surge impact.

### Reflectance analysis: sampling and procedure

All the wood fragments were cleaned of ash particles, incorporated in an epoxy resin and successively polished (See^32,42,45^ for procedure details). The samples were analysed at the Academic Laboratory of Basin Analysis (ALBA) at Roma Tre University, Italy, using a Zeiss Axioskop 40 A pol microscope-photometer system (MPS system) equipped with a tungsten-halogen lamp (12 V, 100 W), an Epiplan- Neofluar 50× oil objective, using filtered 546 nm incident light.

For the calibration of the reflection-photometer mono-crystalline prisms (spinel Ro% = 0.426, sapphire Ro% = 0.595, yttrium-aluminium-garnet Ro% = 0.905 and gadoliniumgallium- garnet (Ro% = 1.726) were used before performing the Reflectance measurements (R2 coefficient equal to or greater than 0.99975).

Based on the fact that the majority of charcoal fragments collected belong to the species Pinus harwegii^70^, we decide to choose among the available five pyrolysis curves, the experimental equation by^41^ because is referred to the same tree genus (e.g., Pinus sylvestris) (See^45^ for details).

### Micro-tomography analysis sampling and procedure

Micro-tomography analysis on wood fragments is a tool broadly used (i.e. in Archeological science^61^; Biosciences^71^; Forest and Wood Science^60^) because of its non-destructive, three-dimensional investigation of wood texture. Micro-tomographic imaging of charcoal fragments results to be a valuable technique for visualizing and quantifying the wood physical properties change caused by thermal degradation (pyrolisys). Studies on forest fires in South Africa^59,60^ and laboratory pyrolysis experiments^61^ have amply demonstrated the usefulness of this method in particular with regard to the characterization of the variations of density, volume, micro-porosity, wood cell diameter and thickness of pine wood subjected to pyrolysis. Starting from this temperature onwards (340–350 °C according to their experiments) wood structure undergoes macroscopic variations depending on temperature degree and exposure time^60^.

Laboratory experiments^60,61^ on raw pine wood demonstrated that increasing temperature during pyrolysis leads to wood volumetric shrinkage, decrease in density and cell wall thickness, increase in cell dimension (lumen diameter) and increase in micro-porosity (increase in pore connectivity).

Depending on the heat exposure time and temperature reached (340–350 °C), wood volume can be reduced at 75% of its original space due to molecular rearrangement and the devolatilization of hemicelluloses^72^. At the same temperature range, cell wall density decreases at 48%, while above 220 °C cell wall become thinner due to loss of oils and liquids. At higher temperature the thinning of wall cell leads to collapse of the wood structure and to an increase in cell dimension and therefore porosity.

Micro-tomography analysis was performed at Laboratorio Universitario de Microtomografía de Rayos X (LUMIR) at UNAM using a CarlZeiss Xradia Versa-510 X-ray μCT, equipped with an X-ray tube with a tungsten anode. The voltage was set to 50 kV and the current of the X-ray source to 83 μA, giving a maximum power of 3 W. No filters were used for the analysis. The detection was performed by an Andor CCD camera with a maximum resolution of 2MP.

Before to proceed in evaluating carbonised fragments micro-porosity, we tasted the Micro-tomographer by evaluating the micro-porosity of raw pine wood first to evaluate the goodness of our data with literature values^70^, and secondly, to have the micro-porosity value of “sample zero” (original raw pine wood, Fig. 4) to compare with the charred wood results. Due to the presence of different wood texture fragment types in our data set, we evaluated the micro-porosity of raw pine cortex and sapwood (inner part) (Fig. 4),

For all the samples the estimation of micro-porosity was performed selecting a cubic representative volume of 400 μm × 400 μm or 300 μm × 300 μm (for smaller samples) in dimensions.

## Data Availability

All data generated or analysed during this study are included in this manuscript. In case of need of further explanations please contact the corresponding author.
